# Region-specific distribution of Olig2-expressing astrocytes in adult mouse brain and spinal cord

**DOI:** 10.1186/s13041-021-00747-0

**Published:** 2021-02-17

**Authors:** Hui Wang, Liang Xu, Chuying Lai, Kaiyu Hou, Junliang Chen, Yaowei Guo, Abhijeet Sambangi, Shreya Swaminathan, Chunming Xie, Zheng Wu, Gong Chen

**Affiliations:** 1 Department of Neurology, Affiliated ZhongDa Hospital, School of Medicine, Southeast University, Nanjing, 210009 Jiangsu China; 2 Department of Biology, Huck Institutes of Life Sciences, Pennsylvania State University, University Park, PA 16802 USA; 3 GHM Institute of CNS Regeneration, Jinan University, Guangzhou, 510632 China; 4 Institute of Neuropsychiatry, Affiliated ZhongDa Hospital, Southeast University, Nanjing, 210009 Jiangsu China

**Keywords:** Olig2, Astrocyte, Oligodendrocyte, Neuron, Central nervous system, Brain, Spinal cord, Microglia

## Abstract

Olig2 is an important transcription factor essential for the specification and differentiation of oligodendrocytes as well as astrocytes and neurons during developmental stages. However, Olig2 distribution pattern and its relationship among different types of glial cells in the adult central nervous system (CNS) are not well characterized. Here, we systematically examined Olig2 expression pattern in combination with major markers of neurons and glial cells throughout the brain and spinal cord in the adult mice. As expected, Olig2 is universally expressed in oligodendrocytes and oligodendrocyte precursor cells (OPCs), but not in neurons or microglia. Interestingly, we discover a subpopulation of Olig2^+^ astrocytes that are highly enriched in some specific regions including the olfactory bulb, thalamus, midbrain, medulla, and spinal cord in the adult mice. Moreover, OPCs have high expression level of Olig2, whereas oligodendrocytes and astrocytes have similar level of Olig2 expression. Our results suggest that a distinct population of Olig2^+^ astrocytes are highly concentrated in discrete regions in the adult CNS. Investigating the functional significance of these Olig2^+^ astrocytes in both resting state and pathological state of the brain and spinal cord may broaden our understanding on astrocytic heterogeneity and functions.

## Introduction

Oligodendrocyte transcription factor 2 (Olig2) is a basic helix-loop-helix (bHLH) transcription factor that is mainly expressed in the CNS [1]. Olig2 regulates neurogenesis primarily as an anti-neurogenic factor but may also be required for certain neuronal differentiation at different stages of development [2, 3]. During embryogenesis, Olig2 first guides the fate of motor neurons by establishing the ventral domain of motor neuron ancestors and promoting neuronal differentiation, and then switches to promoting the formation of OPCs and the differentiation of oligodendrocytes in later stages of development [4–6]. Recent studies have found that Olig2 is expressed in developing astrocytes and plays a pivotal role in astrocyte development in the brain [7–10]. These studies indicate that Olig2 acts as a multifaceted transcription factor for some specific cell type differentiation and maturation during neural development. In the normal adult CNS, Olig2 is continuously expressed in the OPCs, mature oligodendrocytes [11] and a very small percentage of astrocytes (~ 4.3%) [12]. Interestingly, the postnatal deletion of Olig2 will switch OPCs to an astrocyte fate [13]. Olig2 can also be found in reactive astrocytes after brain injury [14] and plays a critical role in reactive astrocyte proliferation and glial scar formation [15]. In general, Olig2^+^ OPCs can differentiate into oligodendrocytes during adulthood [11]. A previous study even reported that OPCs in the piriform cortex can generate some piriform pyramidal neurons in the adult brain [16]. Therefore, Olig2 can be expressed in different cell types and play diversified functions in the adult CNS. However, considering the heterogeneity of cell composition among different CNS regions, the precise Olig2 expression pattern in the adult CNS remains poorly understood.

To systematically investigate the Olig2 expression pattern in the adult mouse CNS, we performed a series of studies on Olig2 expression in the adult CNS by investigating five major subtypes of cells including OPCs, oligodendrocytes, astrocytes, microglia, and neurons. We found that almost all the OPCs and oligodendrocytes express Olig2. No Olig2 signal was detected in microglia or neurons. Interestingly, the expression pattern of Olig2 in adult astrocytes shows region-specific patterns. Most astrocytes in the adult mouse cortex are Olig2-negative. Surprisingly, over 80% of astrocytes are Olig2^+^ in a few regions including the olfactory bulb, midbrain, thalamus, medulla and the spinal cord. Furthermore, the expression level of Olig2 appears to be much higher in OPCs than that in oligodendrocytes and astrocytes. Together, our study provides a holistic view of Olig2 distribution in the adult mouse brain and spinal cord. Our discovery of several discrete CNS regions with high level of Olig2-expression astrocytes suggests that these Olig2^+^ astrocytes may play a distinct function from those Olig2^+^ astrocytes.

## Results

### NG2-expressing cells are only a proportion of Olig2-expressing cells

**Fig. 1:**
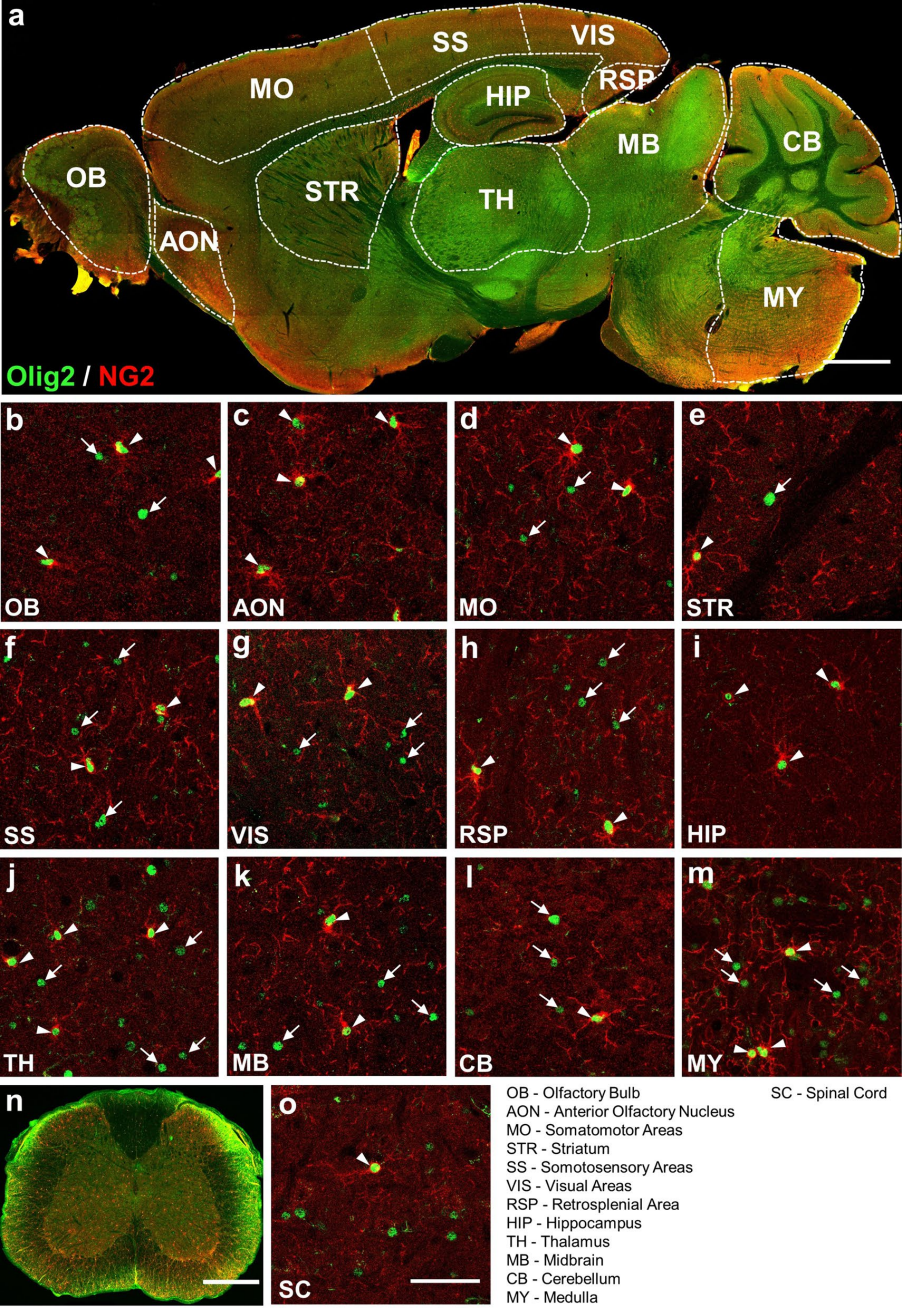
Co-immunostaining of Olig2 and NG2 in adult mouse CNS. **a** Sagittal overview of Olig2 and NG2 expression in the mouse brain. **b**–**m** High magnification images showing the expression relationship between Olig2 and NG2 in 12 selected brain regions. Note that nearly all NG2^+^ cells are co-stained with Olig2 as indicated by white arrowheads, while a considerable number of Olig2^+^ cells do not have NG2 expression, as indicated by white arrows. In the HIP, about 76.1% of Olig2^+^ cells had NG2 co-expression, which is the highest among selected regions. **n** Coronal overview of the mouse SC, showing the co-immunostaining of Olig2 and NG2. **o** High magnification image showing the colocalization of Olig2 and NG2 in the SC of mouse. Note that almost all NG2^+^ cells were positive for Olig2 as in other brain regions, but only 8.3% of Olig2^+^ cells were positive for NG2. Scale bars: 1000 μm (**a**), 50 μm (**b**–**m**, **o**), 500 μm (**n**)

OPCs are a population of glial cells in the CNS which comprise about 5% of total cells and are the main proliferating cell type in the resting state of adult CNS [11]. In the adult CNS, OPCs can differentiate into myelinating oligodendrocytes and are usually characterized as NG2 proteoglycan expressing cells. Therefore, we performed co-immunostaining of Olig2 and NG2 to study their relative distributions in the adult mouse CNS. Figure 1 illustrates the overlay of both Olig2 and NG2 signals across different brain regions (Fig. 1a, sagittal brain section at low magnification) and the spinal cord (SC) (Fig. 1n, coronal section) in an adult mouse. Clearly, while Olig2 and NG2 signal can be detected throughout the CNS regions, their relative expression level is not evenly distributed among different regions. One striking feature is the obvious strong signal of Olig2 (green) in the thalamus (TH) and midbrain (MB) (Fig. 1a). To more clearly understand the relationship between the NG2 and Olig2 expression pattern, we divided the brain into 12 discrete regions including the olfactory bulb (OB), anterior olfactory nucleus (AON), motor cortex (MO), striatum (STR), somatosensory cortex (SS), visual cortex (VIS), retrosplenial area (RSP), hippocampus (Hip), thalamus (TH), midbrain (MB), cerebellum (CB), and medulla (MY) (Fig. 1a). As shown in the high magnification confocal images of the brain (Fig. 1b–m) and SC (Fig. 1o), almost all the NG2-expressing cells in different CNS regions were clearly co-stained with Olig2, suggesting that all NG2 cells express Olig2. However, there were a considerable number of Olig2^+^ cells that did not have NG2, indicating a subpopulation of Olig2-expressing cells that are NG2-negative in the adult CNS. For example, in the thalamus and midbrain, only 13–16% of Olig2^+^ cells were NG2^+^ cells (see Table 1 for quantification). What are the majority of the Olig2^+^ but NG2^−^ cells in the thalamus and midbrain? What about the other CNS regions in the adult mice? To answer these questions, we further conducted a series of experiments to characterize the Olig2^+^ cells throughout various CNS regions.

**Table 1:** The proportion (%) of Olig2-expressing cells corresponding to specific glial cell types in different CNS regions

	Marker			
	CC1^+^	CNPase^+^	NG2^+^	S100β^+^
*Area*				
OB	88.1 ± 2.5	21.0 ± 4.3	32.9 ± 1.4	67.5 ± 4.8
AON	85.1 ± 2.5	20.4 ± 1.9	73.7 ± 8.4	20.1 ± 1.4
MO	94.3 ± 1.2	43.1 ± 8.1	17.6 ± 2.5	13.2 ± 3.5
STR	51.7 ± 9.1	13.3 ± 3.6	61.3 ± 7.9	25.2 ± 3.5
SS	92.2 ± 2.0	69.1 ± 4.2	16.7 ± 1.7	8.5 ± 2.6
VIS	83.0 ± 2.4	72.9 ± 2.7	30.7 ± 1.2	8.8 ± 0.5
RSP	85.1 ± 1.6	82.8 ± 1.4	24.3 ± 1.1	9.6 ± 3.0
DG	66.5 ± 3.9	12.7 ± 7.6	76.1 ± 4.4	2.8 ± 1.9
TH	89.4 ± 1.5	47.8 ± 4.8	13.8 ± 0.9	39.8 ± 1.7
MB	90.9 ± 1.4	84.3 ± 1.5	15.6 ± 2.4	31.6 ± 0.6
CB	97.0 ± 1.3	92.6 ± 0.8	13.8 ± 0.3	6.5 ± 1.2
MY	94.0 ± 1.4	88.1 ± 1.2	13.3 ± 1.7	28.4 ± 2.5
SC	77.0 ± 1.2	89.4 ± 1.2	8.3 ± 0.7	40.6 ± 2.1

The proportion of different cell types in Olig2^+^ cells varied according to different CNS regions (n = 4). In all regions, more than 50% Olig2^+^ cells are CC1-positive. However, the proportion of CNPase^+^ and NG2^+^ cells had a high variation. As for S100β^+^ cells, the proportion in most of the selected regions were below 50% except in the OB (67.5%). Values are presented as mean ± SEM

### Oligodendrocytes are only part of the Olig2-expressing cells

**Fig. 2:**
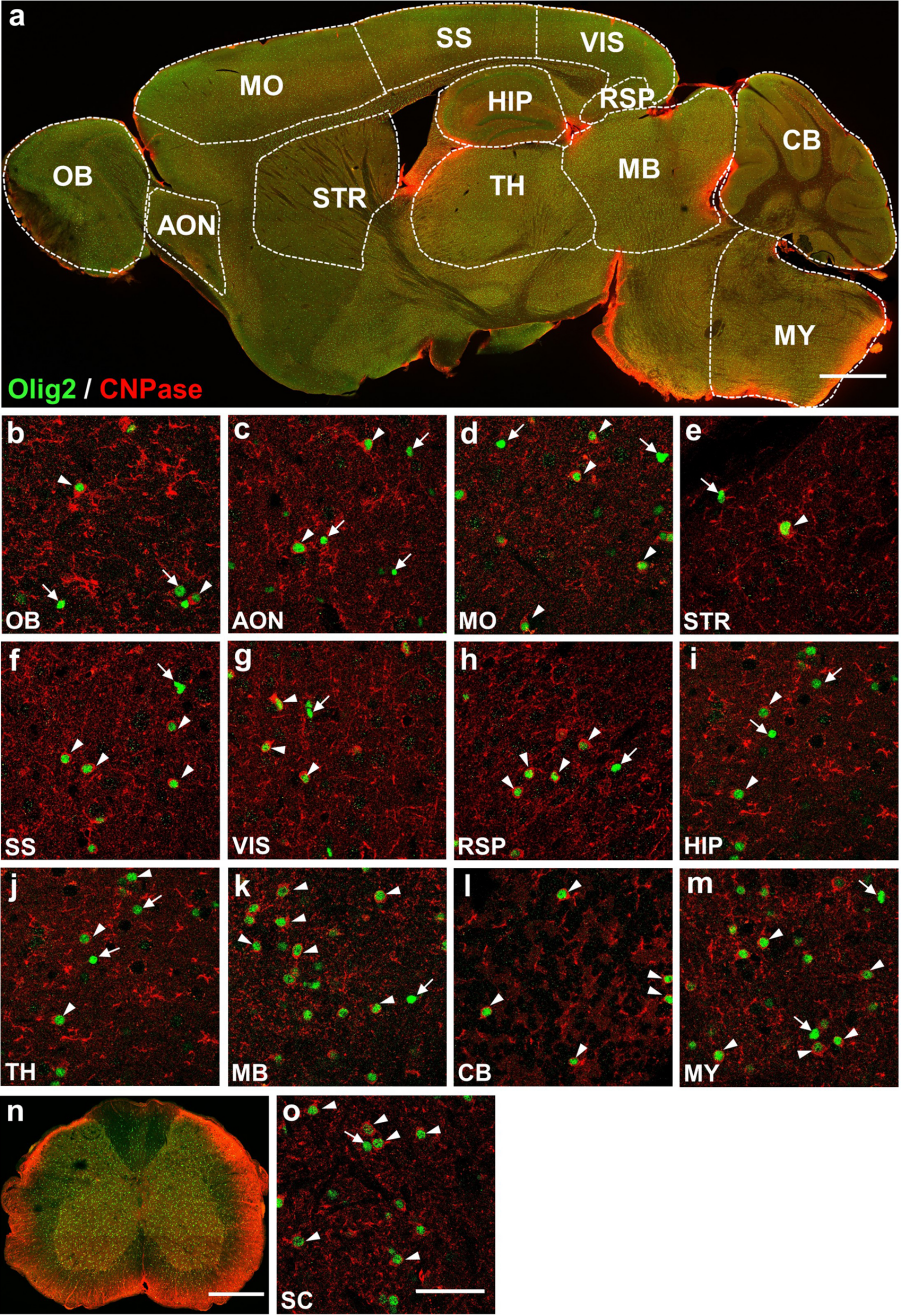
Co-immunostaining of Olig2 and CNPase in adult mouse CNS. **a** Sagittal overview of Olig2 and CNPase expression in the mouse brain. **b**–**m** Representative high magnification images illustrating the expression relationship between Olig2 and CNPase in selected brain regions. All CNPase^+^ cells are co-stained with Olig2 as indicated by white arrowheads. On the other hand, Olig2^+^ cells that are not co-stained with CNPase are indicated by white arrows. The proportion of the CNPase and Olig2 double positive cells in total Olig2^+^ cells ranges from 12.7% to 92.6% among selected regions. **n** Coronal overview of the mouse SC, showing co-immunostaining of Olig2 and CNPase. **o** High magnification image showing the co-immunostaining of Olig2 and CNPase in the SC of mouse. Similar to the brain, almost all CNPase^+^ cells were positive for Olig2. On the contrary, about 89.4% of Olig2^+^ cells were co-labelled with CNPase. Scale bars: 1000 μm (**a**), 50 μm (**b**–**m**, **o**), 500 μm (**n**)

Oligodendrocytes are myelinating cells that are differentiated from OPCs under the control of bHLH transcriptional factor Olig2 [17]. Besides OPCs, we also analyzed the expression pattern of Olig2 in oligodendrocytes by co-staining Olig2 with two typical markers of oligodendrocytes, CNPase and CC1 [18]. CNPase, comprising about 4% of the total myelin protein in the CNS, is widely expressed in pre-myelinating and myelinating oligodendrocytes [19]. We detected CNPase expression in the whole brain (Fig. 2a) and spinal cord (Fig. 2n), reflecting a uniform distribution of oligodendrocytes in the adult CNS. At high magnification, we found that all the CNPase-expressing oligodendrocytes had Olig2 immunofluorescence signal (Fig. 2b–m, o). However, not every Olig2^+^ cells showed CNPase signal (Fig. 2b–m, o, Table 1), indicating the diversification of the proportion of oligodendrocytes in the adult CNS.

**Fig. 3:**
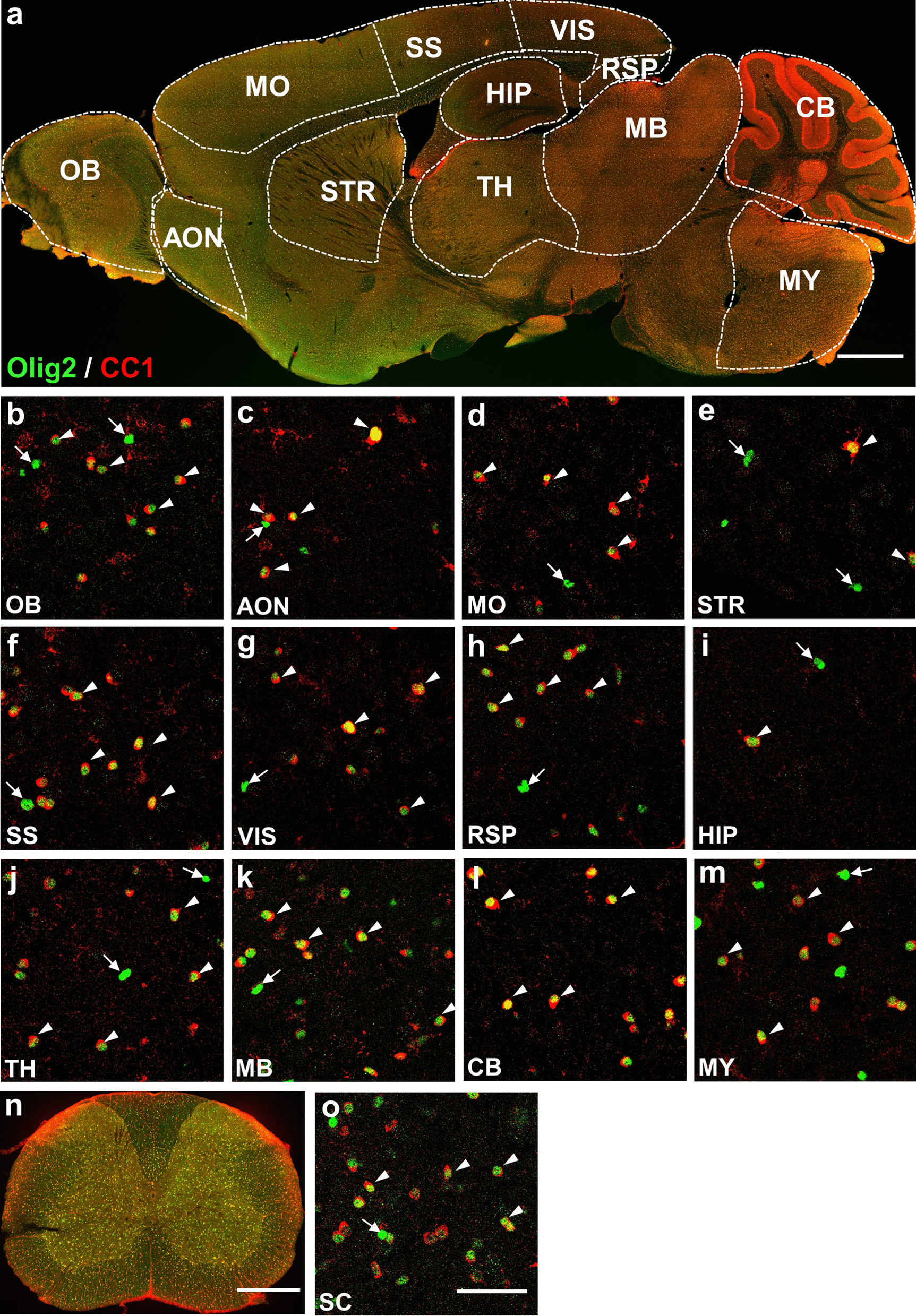
Co-immunostaining of Olig2 and CC1 in adult mouse CNS. **a** Sagittal overview of Olig2 and CC1 expression pattern in the mouse brain. **b**–**m** High magnification images showing the expression relationship between Olig2 and CC1 in brain regions of interest. Most of the CC1^+^ cells have Olig2 immuno signal as indicated by white arrowheads. On the other hand, white arrows indicate Olig2^+^ cells without CC1. The proportion of CC1^+^ and Olig2^+^ cells is about 50% in the STR and HIP, and higher than 80% in other brain regions. **n** Coronal overview of the mouse SC, showing co-immunostaining of Olig2 and CC1. **o** High magnification image displaying the co-immunostaining of Olig2 and CC1 in the SCe. As in the brain regions, the majority of the CC1^+^ cells are positive for Olig2 (white arrowheads), quantified data showing about 77.0% of Olig2^+^ cells are CC1^+^ (white arrows). Scale bars: 1000 μm (**a**), 50 μm (**b**–**m**, **o**), 500 μm (**n**)

Next, we performed co-immunostaining of Olig2 with mature oligodendrocyte marker CC1. Unlike myelin protein CNPase, CC1 was highly localized in the soma of the oligodendrocytes (Fig. 3). Our data showed that a majority of CC1^+^ cells (red) were co-stained with Olig2 (green) throughout the brain (91.0% ~ 99.6%, white arrowheads, Fig. 3b, d–m) and spinal cord (98.70%, white arrowheads, Fig. 3n). On the other hand, while most brain regions showed high colocalization between Olig2 and CC1, we also found Olig2^+^ but CC1^−^ cells in some CNS regions (Fig. 3b–d, 3f–o, and Table 1). Particularly, in the striatum only 51.7% of Olig2^+^ cells showed CC1 signal, suggesting that many Olig2^+^ cells are not oligodendrocytes in the striatum (Fig. 3e and Table 1). Together, these data suggest that there is a subpopulation of Olig2^+^ cells in some adult CNS regions that are neither oligodendrocytes nor OPCs.

### Olig2 not detected in neurons and microglia

**Fig. 4:**
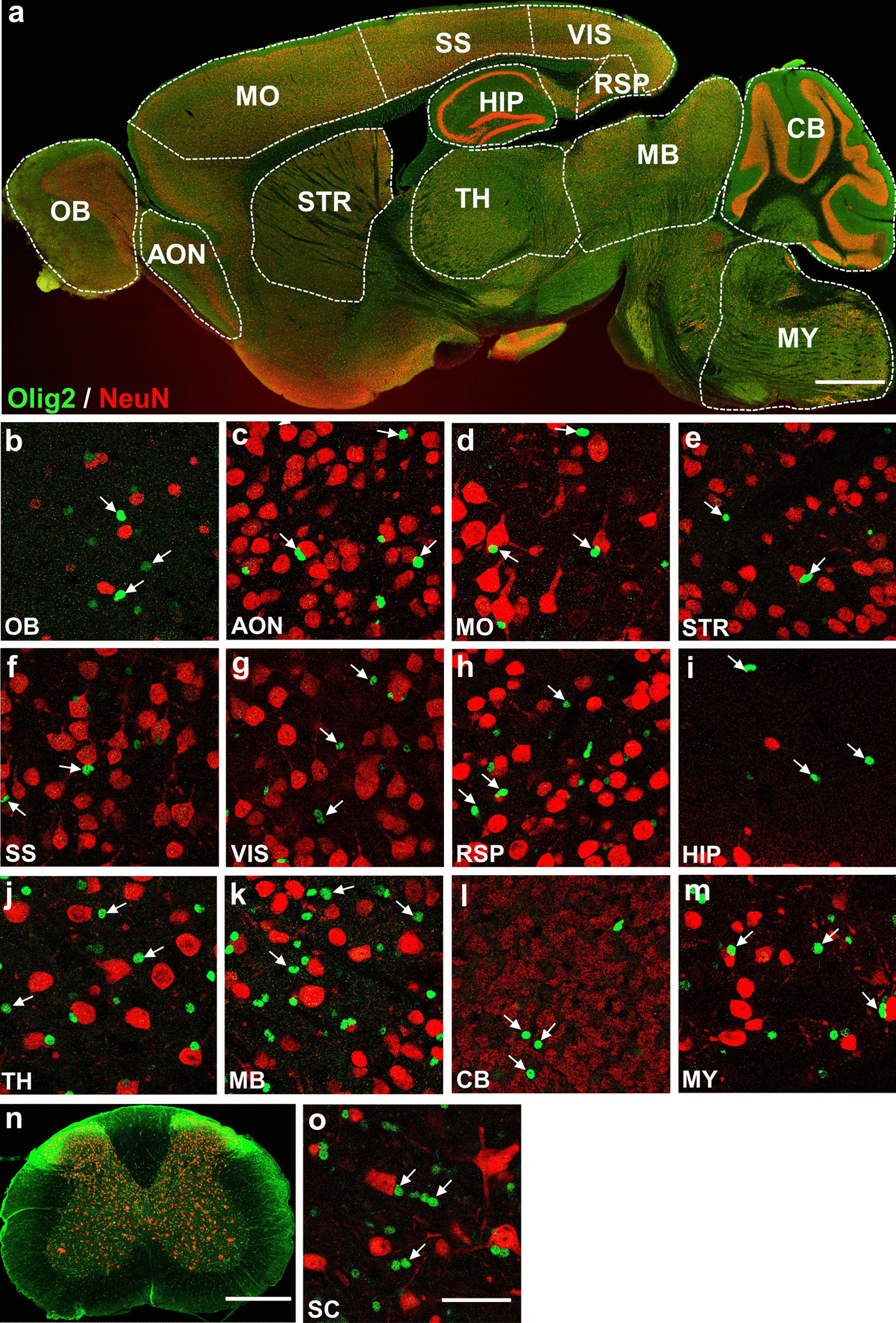
Co-immunostaining of Olig2 and NeuN in adult mouse CNS. **a** Sagittal overview of Olig2 and NeuN expression pattern in the mouse brain. **b**–**m** High magnification images showing the expression relationship between Olig2 and NeuN in selected brain regions. Co-localization of Olig2 and NeuN is not observed in all the regions of interest as indicated by white arrows. **n** Coronal overview of the mouse SC. **o** A high magnification image depicting the co-immunostaining of Olig2 and NeuN in the SC. Similarly, no Olig2 is expressed in NeuN^+^ cells. White arrows indicate Olig2^+^ cells without NeuN co-localization. Scale bars: 1000 μm (**a**), 50 μm (**b**–**m**, **o**), 500 μm (**n**)

**Fig. 5:**
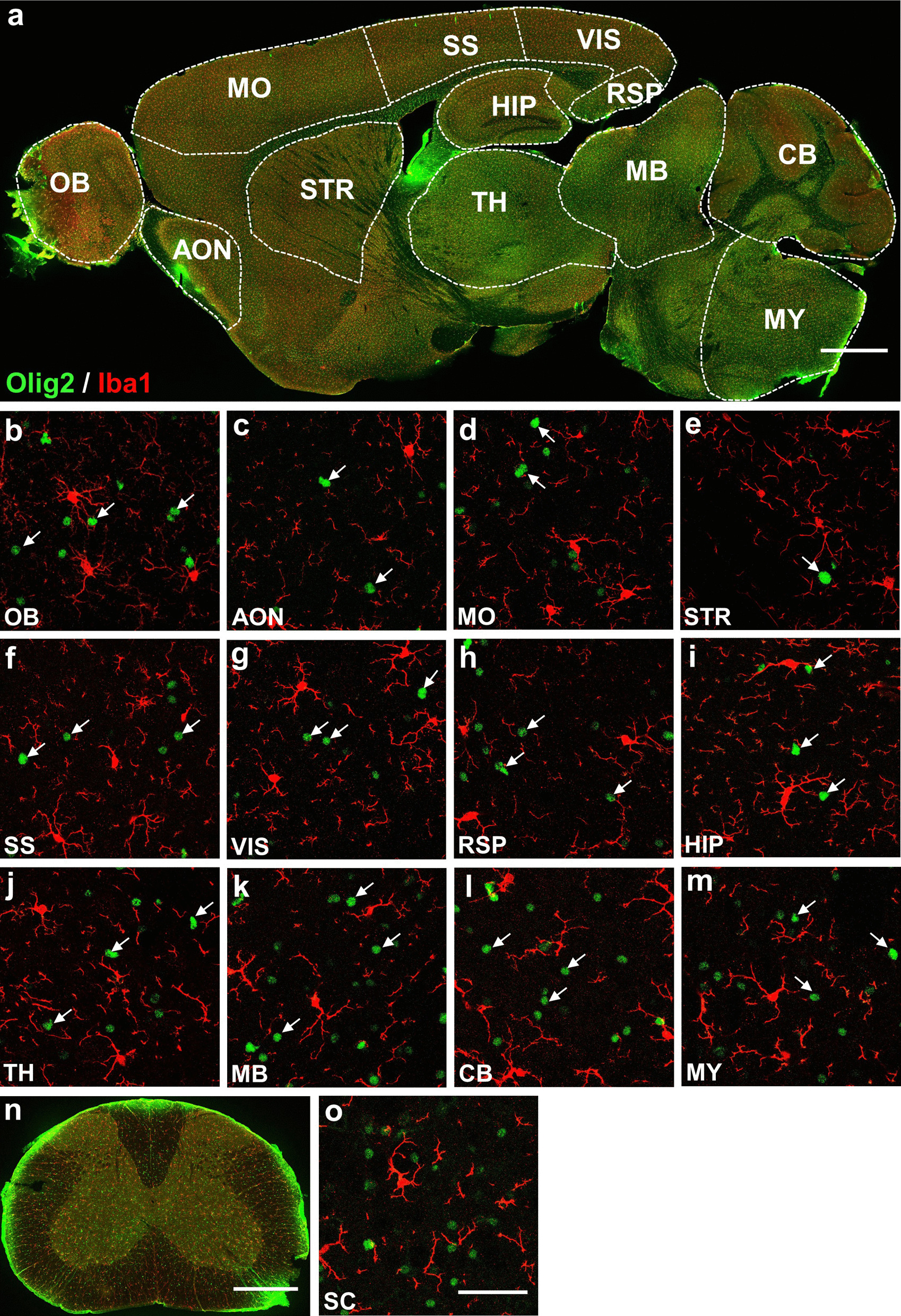
Co-immunostaining of Olig2 and Iba1 in adult mouse CNS. **a** Sagittal overview of Olig2 and Iba1 expression in the mouse brain. **b**–**m** High magnification images showing the expression relationship between Olig2 and Iba1 in selected brain regions. There is no co-localization of Olig2 and Iba1 in all the regions of interest as indicated by white arrows. **n** Coronal overview of the mouse SC. **o** A high magnification image presenting there is no co-immunostaining of Olig2 and Iba1 in the SC (white arrows). Scale bars: 1000 μm (**a**), 50 μm (**b**–**m**, **o**), 500 μm (**n**)

Neurons are electrically excitable cells that are the basic functional units of the CNS. Olig2 plays a crucial role in the generation of motor neurons in the developing spinal cord. We investigated whether Olig2 could be continuously expressed in some neurons of the adult CNS. However, we did not find any NeuN^+^ neurons that co-immunolabeled with Olig2 in either the brain or spinal cord (Fig. 4). Furthermore, we also performed co-immunostaining of Olig2 with microglia marker Iba1 and did not detect any Olig2^+^ cells co-expressing Iba1 (Fig. 5). These data indicate that both neurons and microglia do not express Olig2 in the adult mouse CNS.

### Region-specific expression of Olig2 in astrocytes

**Fig. 6:**
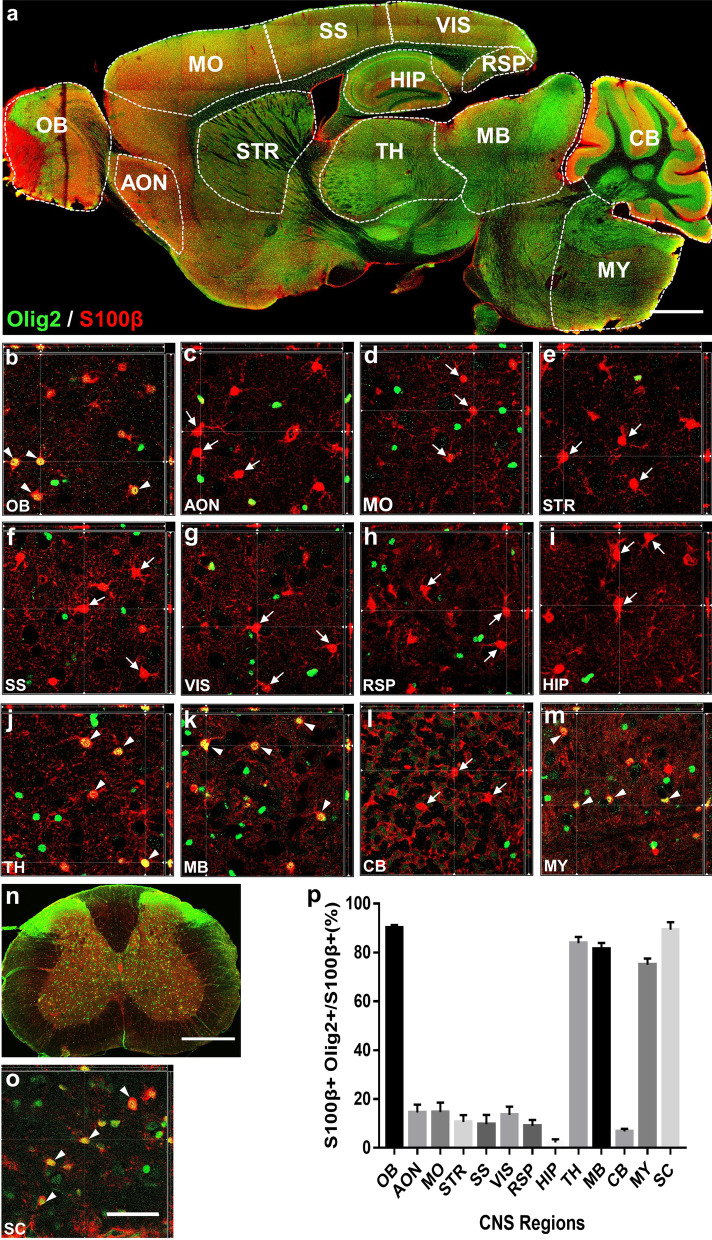
The percentage of Olig2^+^ astrocytes in different mouse CNS regions. **a** Sagittal overview of the mouse brain indicating a variety of brain regions of interest. **b**–**m** High magnification images showing the olig2 and S100β double immune-staining in different mouse brain regions. White arrowheads indicate co-localization of Olig2 and S100β, while white arrows indicate astrocytes that do not express Olig2. Note that in the AON (**c**), STR (**e**), HIP (**i**), CB (**l**) and the cortical areas including the MO (**d**), SS (**f**), VIS (**g**) and the RSP (**h**), few astrocytes expresse Olig2, while in the OB (**b**), TH (**j**), MB (**k**), and the MY (**m**), majority astrocytes (> 70%) have Olig2 immune reactivity. **n** Coronal overview of the mouse SC showing Olig2^+^ astrocytes. **o** High magnification image displaying the co-localization of Olig2 and S100β in the SC. Note that most of the astrocytes (89.3%) in the SC expresse Olig2. **p** The ratio of Olig2^+^ cells in total of the S100β^+^ cells in each brain region and the SC. Data are represented as mean ± SEM. Scale bars: 1000 μm (**a**), 50 μm (**b**–**m**, **o**), 500 μm (**n**)

Astrocytes are resident glial cells in the CNS besides oligodendrocytes and microglia. We further investigated whether Olig2 expression can be detected in the astrocytes of adult CNS by performing co-immunostaining of Olig2 with S100β, an astrocyte specific marker. As shown in the tiled Fig. 6a, S100β signal was found throughout the adult CNS, with very high level immune reactivity detected in the OB, AON, MO, and the molecular layer of the cerebellum. Interestingly, high magnification confocal images found that many S100β^+^ astrocytes also expressed Olig2 in a region-specific pattern. In the AON, STR, HIP, CB (white arrows, Fig. 6c, e, i, l) and cortical areas including the MO, SS, VIS and the RSP (white arrows, Fig. 6d, f–h), very few Olig2 was located within astrocytes (quantified in Fig. 6p). Unexpectedly, we found a large number of S100β^+^ astrocytes that were also Olig2-positive in the OB, TH, MB, and MY (white arrowheads, Fig. 6b, j, k, m). Quantified data showed that the majority of astrocytes (> 70%) in these regions co-expressed Olig2 (Fig. 6p). An even higher proportion of S100β^+^ astrocytes co-expressed Olig2 that were detected in the adult mouse spinal cord (89.3%, Fig. 6n–p). We also calculated the percentage of S100β^+^ and Olig2^+^ astrocytes among the total Olig2^+^ cells, and found that in some areas such as the OB, TH, MB, MY and SC, more than a quarter of Olig2^+^ cells are astrocytes (Table 1). Therefore, our results suggest that there is a distinct subpopulation of astrocytes with a high level of Olig2 expression in some specific regions in the adult mouse CNS.

To further confirm that astrocytes have Olig2 in some specific CNS regions, we performed co-immunostaining of Olig2 with another astrocytic marker Sox9 [20]. Similarly, we detected a significant number of Sox9^+^ cells that were also Olig2-positive in the TH, MB, MY and SC (white arrowheads, Additional file 1: Figure S1j, k, m and o). Quantified data showed that over half of Sox9^+^ cells also expressed Olig2 in the TH, MB, MY and SC. Especially, over 90% of Sox9^+^ cells in the spinal cord were also Olig2-expressing cells. Together, our results further confirm that astrocytes can express Olig2 in some regions of adult mouse brain and spinal cord.

### Highest Olig2 expression level in OPCs

**Fig. 7:**
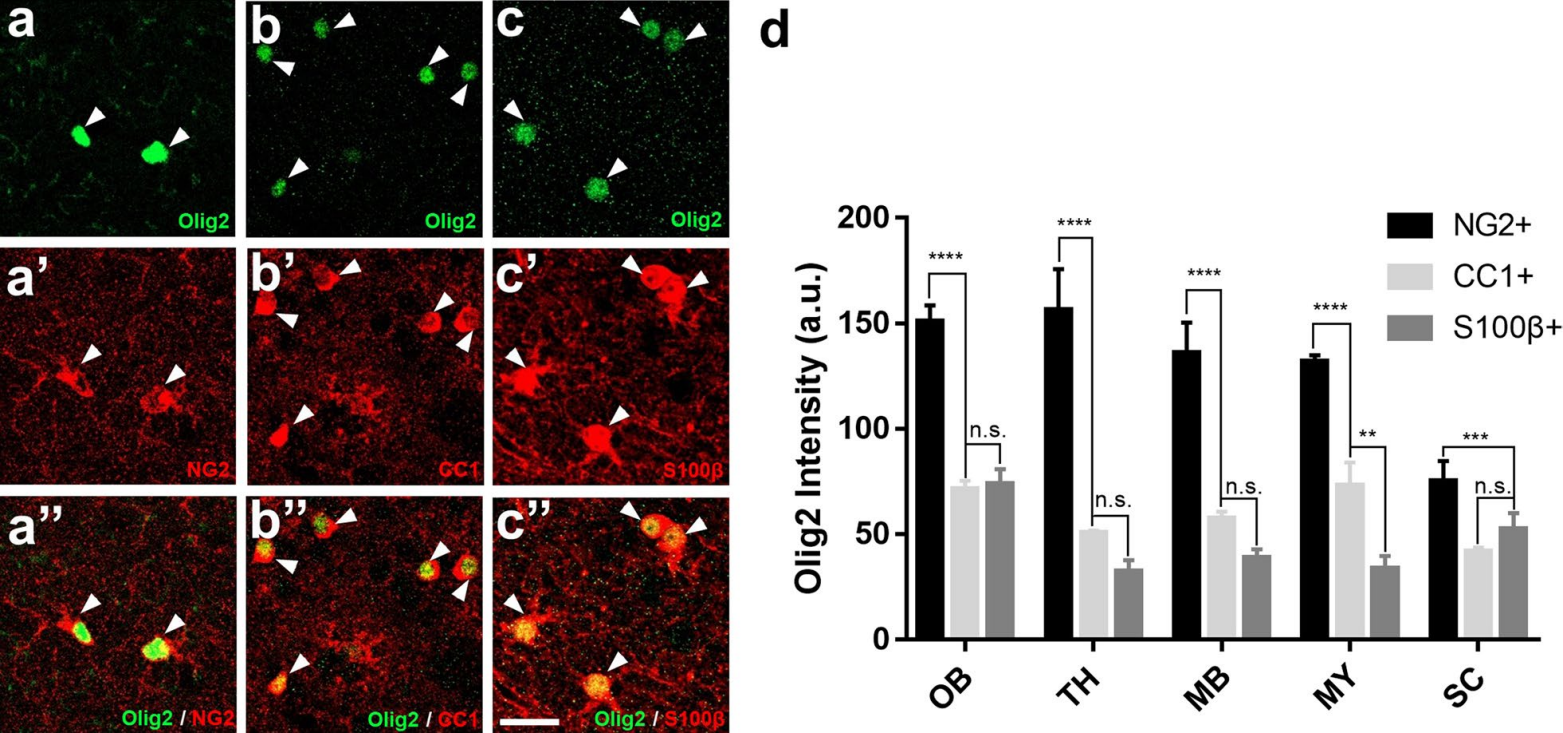
Comparison of Olig2 fluorescence intensity in OPCs, oligodendrocytes and astrocytes. **a**–**c**, **a’**–**c’**, **a’’**–**c’’** Taking the olfactory bulb as an example, the Olig2 signal intensity in NG2^+^ OPCs is much stronger than that in CC1^+^ oligodendrocytes and S100β^+^ astrocytes. However, there is no significant difference of Olig2 intensity between oligodendrocytes and astrocytes. **d** Comprehensive quantitative comparison of fluorescence intensity of Olig2 in OPCs, oligodendrocytes, and astrocytes in the five CNS regions where Olig2^+^ astrocytes are enriched. OPCs has the strongest Olig2 signal. In TH, MB, and MY, there is no significant difference of olig2 signal between oligodendrocytes and astrocytes, but it has significant difference in MY region. In SC, no significant difference was detected between oligodendrocytes and astrocytes. Scale bars: 20 μm (**a**–**c**, **a’**–**c’**, **a’’**–**c’’**). All values are presented as mean ± SEM; One-way ANOVA test; **p < 0.01, ***p < 0.001, ****p < 0.0001

After discovering remarkable Olig2 expression in astrocytes within some specific regions, we further investigated whether the Olig2 expression level in astrocytes is comparable to that of OPCs and oligodendrocytes. When compared side-by-side among astrocytes, OPCs, and oligodendrocytes, we found that OPCs expressed much higher level of Olig2 signal than that of oligodendrocytes and astrocytes in the OB (Fig. 7a–c), and TH, MB, and MY, as well as the spinal cord (Fig. 7d,  p< 0.001). Interestingly, the Olig2 expression level was comparable between oligodendrocytes and astrocytes (Fig. 7). These results indicate that in some regions of the adult mouse CNS under normal conditions, astrocytes can express Olig2 at a similar level to the oligodendrocytes.

## Discussion

In this study, we discovered that Olig2 was expressed not only in OPCs and oligodendrocytes but also in some astrocytes in the adult mouse CNS. Most intriguingly, the Olig2-expressing astrocytes showed clear regional specificity with high enrichment in certain regions such as OB, STR, TH, MB, MY, and spinal cord. Such distinct subpopulation of Olig2^+^ astrocytes underscores the heterogeneity of astrocytes and implies potential functional divergence from Olig2^−^ astrocytes.

### Astrocyte origination and heterogeneity

During gliogenesis, both astrocytes and oligodendrocytes originate from radial glial cells that are derived from neuroepithelial cells [3, 21]. Different subtypes of astrocytes may be dependent on the differential combination of SLIT1 and Reelin under the regulation of different transcription factors activated by SHH signaling [3]. Interestingly, in the mouse spinal cord, a subpopulation of Olig2^+^ astrocyte progenitor cells are reported to be produced in the same region where oligodendrocytes are generated [7, 10], which might explain why we observed a large number of Olig2^+^ astrocytes in the adult spinal cord. Besides produced directly from the transformation of radial glia cells, astrocytes can also be produced from the proliferation of differentiated astrocytes [3, 22, 23]. Therefore, both embryonic and postnatal astrogliosis contribute to the complex origination of astrocytes. It will be interesting to trace back where the Olig2^+^ astrocytes are generated: the embryonic radial glia or postnatal astrocytes.

Astrocytes are ubiquitous in the adult CNS, playing key roles in structural and trophic support, including blood–brain-barrier formation, ion homeostasis, and synaptic transmission [24]. Ramón y Cajal’s seminal work identified two classic groups of astrocytes: fibrous astrocytes in the white matter and protoplasmic astrocytes in the gray matter. Recent studies demonstrate that astrocytes may have region-specific characteristics in morphology [25], gene expression [26] and function [27]. Our study here discovers that Olig2, an oligodendroglia-specific marker, is highly expressed in certain astrocytes within some discrete CNS regions, suggesting that Olig2 might be an important regulator of astrocyte heterogeneity. Previously, Xin et al. [28] reported that glutamine synthetase (GS), an astrocyte-specific marker, is highly expressed in oligodendrocytes in the midbrain and spinal cord. Combined with our finding of a region-dependent Olig2 expression in astrocytes, these results together further suggest that astrocytes and oligodendrocytes may share certain common properties, at least in certain regions. In our present study, we found that the Olig2 expression level in astrocytes is comparable to that in CC1-labeled mature oligodendrocytes, but not at the level of OPCs. Future studies may help us to better understand the relationship between Olig2^+^ astrocytes and GS^+^ oligodendrocytes in the adult CNS.

### Olig2 and astrocyte fate

Olig2 has been reported to play a critical role not only in the specification and differentiation of oligodendroglia, but also in regulating astrocytic fate during early neural development. Lineage tracing studies found that a subset of Olig2^+^ progenitor cells can differentiate into astrocytes, accompanied by downregulation of Olig2 [7, 29]. In neuroepithelial cells, knockout of Olig2 resulted in the differentiation of astrocytes but not oligodendrocytes [30], whereas overexpression of Olig2 inhibited the generation of astrocytes [31]. In vivo studies in prenatal and postnatal mice have also found that forced inhibition of Olig2 is sufficient for the fate switch from oligodendroglia to astrocytes [29], suggesting that Olig2 may function as a switch to turn glial fate toward astrocytes or oligodendrocytes. Interestingly, a subpopulation of S100β^+^ astrocytes have been clearly labeled in Olig2-transgenic mice [32]. Conversely, a subpopulation of oligodendrocytes and OPCs are also clearly labeled in S100β-transgenic mice [33, 34]. Our studies reveal that there are widespread Olig2^+^ astrocytes throughout the brain and spinal cord, and in certain regions the ratio of Olig2^+^ astrocytes can be very high such as that in the OB with 67.5% being Olig2^+^ astrocytes. Therefore, while Olig2 is a key factor favoring the fate-determination of oligodendroglia, some glial cells appear to adopt astrocytic fate regardless the presence of Olig2.

### Olig2 and astrocyte function

Under brain injury and disease conditions such as Alzheimer’s disease and ischemic stroke, Olig2 is robustly upregulated in reactive astrocytes [14, 15, 35]. On the other hand, Olig2 ablation in astrocytes dramatically reduces the formation of reactive astrocytes as well as their proliferation [15] while improving endogenous neurogenesis [35, 36]. Since reactive astrocytes play a protective role in response to injury, the upregulation of Olig2 in reactive astrocytes may be involved in the mechanism of early CNS self-defense in adult animals. However, in later stage of injury or disease, Olig2^+^ reactive astrocytes may be detrimental to neural recovery. Our current finding of Olig2^+^ astrocytes in the resting state of adult CNS certainly raises an important question of what is the function of Olig2 in these differentiated adult astrocytes? Since Olig2 is a bHLH transcription factor, it can form dimer with other bHLH factors including neural transcription factors such as NeuroD1 and neurogenin-2 (Ngn2). Therefore, one speculation of the Olig2 function in the adult astrocytes is an inhibitory function to prevent neurogenesis due to accidental activation of neural transcription factors. Another possibility is that since Olig2 can directly bind to chromosomal DNA and regulate the expression of many downstream genes, including both neuronal and glial genes, the presence of Olig2 in the adult astrocytes is an assurance that neuronal genes will be constantly inhibited. Another hypothesis is that these Olig2^+^ astrocytes are more sensitive to environmental changes, such as injury and disease, and therefore will respond rapidly to various stimulations in order to provide adequate protections to the surrounding cells. It will be important for future studies to dissect out the precise function of these Olig2^+^ astrocytes in different disease states such as Alzheimer’s disease and stroke.

Here, we have identified a large population of mature astrocyte that maintain expression of Olig2 in the adult mouse CNS, this observation raises the question of whether the expression of Olig2 confers specific functional properties to Olig2 expression astrocytes, that needs further study in the future.

## Conclusion

This study presents a comprehensive Olig2 expression pattern in the adult mouse CNS, and identifies a subtype of Olig2^+^ astrocytes that are regionally enriched in the brain and spinal cord.

## Methods

### Animals

Wild-type, 5-month-old C57BL/6 mice were used for most of the experiments and analysis. Both male (n = 2) and female mice (n = 2) were used in this study. The mice were housed in a 12/12-h light–dark cycle room and received food and water ad libitum. Experimental protocols were approved by the Pennsylvania State University IACUC, and procedures were performed in line with the guidelines of the National Institute of Health (NIH).

For Sox9 and Olig2 immunostaining study, the 3-month-old C57BL/6 mice were used for the analysis (total 4 mice, 2 male and 2 female). The mice were housed in a 12 h light/dark cycle and supplied with enough food and water. Experimental protocols were approved by Jinan University IACUC.

### Immunohistochemistry and analysis

Mice were anesthetized with Avertin (tribromoethanol, 250 mg/kg, i.p.) and then quickly transcardially perfused with ice-cold artificial cerebrospinal fluid (aCSF). The brain and spinal cord were removed and post-fixed in 4% PFA overnight in darkness at 4℃. The spinal cord was then dehydrated in 30% sucrose for another day. The brain sample was sagittally sectioned at 40 μm with a vibratome (Leica, VTS1000), and the spinal cord was coronally sectioned at 40 μm with a cryostat (Leica, CM1950), then slices were stored in 0.1 M PB at 4 °C. Both brain and spinal cord slices were rinsed three times with PBS for ten minutes per rinse. Slices were blocked with 0.3% triton PBS + 5% normal donkey serum (NDS) for two hours at room temperature. Slices were incubated with primary antibodies diluted in 0.05% triton PBS + 5% NDS in a moist environment for two nights in darkness at 4℃. Then slices were rinsed three times with PBS for ten minutes per rinse and incubated with secondary antibodies diluted in 0.05% triton PBS + 5% NDS for two hours at room temperature. After three times of washing using PBS, the slices were mounted on glass slides with anti-fading solution. For double immunostaining, slices were simultaneously incubated with Olig2 respectively with S100β, CC1, CNPase, NG2, NeuN, or Iba1. The following primary antibodies were used for immunohistochemistry: rabbit anti-Olig2 (Millipore, 1:1000), mouse anti-S100β (Abcam, 1:1000), mouse anti-CC1 (Abcam, 1:300), mouse anti-CNPase (Abcam, 1:1000), mouse anti-Olig2 (Millipore, 1:1000), rabbit anti-NG2 (Millipore, 1:200), guinea pig anti-NeuN (Millipore, 1:2000), rabbit anti-Iba1 (Wako, 1:1000). All secondary antibodies were Alexa Fluor-conjugated from Jackson ImmunoResearch that included donkey anti-rabbit 488, donkey anti-mouse 594, donkey anti-mouse 488, donkey anti-rabbit-594, donkey anti-guinea pig 647, donkey anti-rabbit 594.

The images were acquired by Olympus FV1200 and Zeiss confocal microscope (LSM 800). For quantification in each CNS region of interest, at least 4 areas of 40 × lens were randomly selected and imaged. The white matter cells of mouse CNS were not involved in this study. The quantification of Olig2 intensity was completed by the Zeiss software ZEN. The intensity indicates the mean gray value: mean gray value = integrated signal density/area.

### Statistical analysis

The comparison of Olig2 fluorescence intensity among groups was analyzed by One-Way ANOVA test. Data were presented as mean ± standard mean of error (SEM). GraphPad Prism software was used for data analysis and figure generation. Significance was set at the threshold: *p < 0.05; **p < 0.01; ***p < 0.001, ****p < 0.0001.

## Supplementary Information

**Additional file 1: Table S1.** Zeiss Confocal imaging acquisition parameters. **Table S2.** The proportion (%) of S100β^+^ and Sox9^+^ cells with Olig2 signal in different CNS regions. **Figure S1.** The percentage of SOX9^+^ cells co-labeled with Olig2 in different adult mouse CNS regions.

## Data Availability

The datasets used and/or analysed during the current study are available from the corresponding author on reasonable request.

## Abbreviations

CNS: Central nervous system
OPCs: Oligodendrocyte precursor cells
Olig2: Oligodendrocyte transcription factor 2
bHLH: Basic helix-loop-helix
SC: Spinal cord
TH: Thalamus
MB: Midbrain
OB: Olfactory bulb
AON: Anterior olfactory nucleus
MO: Motor cortex
STR: Striatum
SS: Somatosensory cortex
VIS: Visual cortex
RSP: Retrosplenial area
Hip: Hippocampus
CB: Cerebellum
MY: Medulla
GS: Glutamine synthetase
NIH: National Institute of Health
aCSF: Artificial cerebrospinal fluid
NDS: Normal donkey serum
SEM: Standard mean of error
