# Supplementary material for: Region-specific distribution of Olig2-expressing astrocytes in adult mouse brain and spinal cord
Source: Mol Brain. 2021 Feb 17;14:36. doi: 10.1186/s13041-021-00747-0 (PMC7901088; doi:10.1186/s13041-021-00747-0)
Supplement: Supplementary file 1 — Additional file 1: Table S1. Zeiss Confocal imaging acquisition parameters. Table S2. The proportion (%) of S100β+ and Sox9+ cells with Olig2 signal in different CNS regions. Figure S1. The percentage of SOX9+ cells co-labeled with Olig2 in different adult mouse CNS regions. [file 13041_2021_747_MOESM1_ESM.docx]

**Supplementary Info for:**

**Region-specific distribution of Olig2-expressing astrocytes in adult mouse brain and spinal cord**

Hui Wang^1,2^, Liang Xu^3^, Chuying Lai^3^, Kaiyu Hou^3^, Junliang Chen^3^, Yaowei Guo^3^, Abhijeet Sambangi^2^, Shreya Swaminathan^2^, Chunming Xie^1,4*^, Zheng Wu^2,3*^, Gong Chen^2,3*^

^1^Department of Neurology, Affiliated ZhongDa Hospital, School of Medicine, Southeast University, Nanjing, Jiangsu, China, 210009

^2^Department of Biology, Huck Institutes of Life Sciences, Pennsylvania State University, University Park, PA 16802, USA

^3^GHM Institute of CNS Regeneration, Jinan University, Guangzhou 510632, China

^4^Institute of Neuropsychiatry, Affiliated ZhongDa Hospital, Southeast University, Nanjing, Jiangsu, China, 210009

*Correspondence should be addressed to:

Chunming Xie, M.D. & Ph.D., Department of Neurology, Institute of Neuropsychiatry, Affiliated Zhongda Hospital, School of Medicine, Southeast University, Nanjing, China, 210009;

Tel: 0086-25-83262241

E-mail: [chmxie@163.com](mailto:chmxie@163.com)

Or

Zheng Wu, Ph.D., Professor

GHM Institute of CNS Regeneration, Jinan University

Guangzhou 510632, China

Tel: (86)020-85228384

Email: zhengwu@jnu.edu.cn

Or

Gong Chen, Ph.D., Professor

GHM Institute of CNS Regeneration, Jinan University

Guangzhou 510632, China

Tel: (86) 156-2647-4467

Email: [gongchen@jnu.edu.cn](mailto:gongchen@jnu.edu.cn)

**Supplementary Table 1. Zeiss Confocal imaging acquisition parameters**

| **Zeiss confocal microscope LSM 800** | **Protein markers** | **Laser Wavelength** | **Detector Gain** | **Detector Digital Gain** |
| --- | --- | --- | --- | --- |
| Fig 1b-m, o, Fig 2 b-m, o, Fig 3 b-m, o, Fig 4 b-m, o, Fig 5 b-m, o, Fig 6 b-m, o, Fig 7 a-c, a’’-c’’ | Olig2 | 488nm: 1.00% | 700v | 1 |
| Fig 1b-m, o, Fig 2 b-m, o, Fig 3 b-m, o, Fig 4 b-m, o, Fig 5 b-m, o, Fig 6 b-m, o, Fig 7 a’, a’’ | NG2 | 640nm: 1.20% | 750 | 1 |
| Fig 1b-m, o, Fig 2 b-m, o, Fig 3 b-m, o, Fig 4 b-m, o, Fig 5 b-m, o, Fig 6 b-m, o | CNPase | 640nm: 1.50% | 750 | 1 |
| Fig 1b-m, o, Fig 2 b-m, o, Fig 3 b-m, o, Fig 4 b-m, o, Fig 5 b-m, o, Fig 6 b-m, o, Fig 7 b’, b’’ | CC1 | 640nm: 1.90% | 660 | 1 |
| Fig 1b-m, o, Fig 2 b-m, o, Fig 3 b-m, o, Fig 4 b-m, o, Fig 5 b-m, o, Fig 6 b-m, o | NeuN | 640nm: 5.00% | 800 | 1 |
| Fig 1b-m, o, Fig 2 b-m, o, Fig 3 b-m, o, Fig 4 b-m, o, Fig 5 b-m, o, Fig 6 b-m, o | Iba1 | 640nm: 1.90% | 600 | 1 |
| Fig 1b-m, o, Fig 2 b-m, o, Fig 3 b-m, o, Fig 4 b-m, o, Fig 5 b-m, o, Fig 6 b-m, o, Fig 7 c’, c’’ | S100β | 640nm: 1.00% | 660 | 1 |

**Supplementary Table 2. The proportion (%) of S100β^+^ and Sox9^+^ cells with Olig2 signal in different CNS regions**

|  | S100β^+^ | Sox9^+^ |
| --- | --- | --- |
| OB | 90.1 ± 1.2 | 20.8 ± 5.5 |
| AON | 14.5 ± 3.2 | 35.3 ± 6.2 |
| MO | 14.6 ± 3.9 | 31.3 ± 1.7 |
| STR | 10.6 ± 2.9 | 14.3 ± 4.2 |
| SS | 9.7 ± 3.8 | 27.5 ± 1.3 |
| VIS | 13.5 ± 3.4 | 29.0 ± 4.2 |
| RSP | 9.0 ± 2.4 | 19.0 ± 1.8 |
| HIP | 2.2 ± 1.3 | 10.8 ± 1.5 |
| TH | 83.7 ± 2.6 | 71.0 ± 9.3 |
| MB | 81.5 ± 2.4 | 54.0 ± 8.2 |
| CB | 6.7 ± 1.1 | 25.5 ± 5.3 |
| MY | 75.0 ± 2.5 | 69.8 ± 12.8 |
| SC | 89.3 ± 3.1 | 93.3 ± 2.1 |

**Supplementary Figure 1.** **The percentage of SOX9^+^ cells co-labeled with Olig2 in different adult mouse CNS regions.**

**
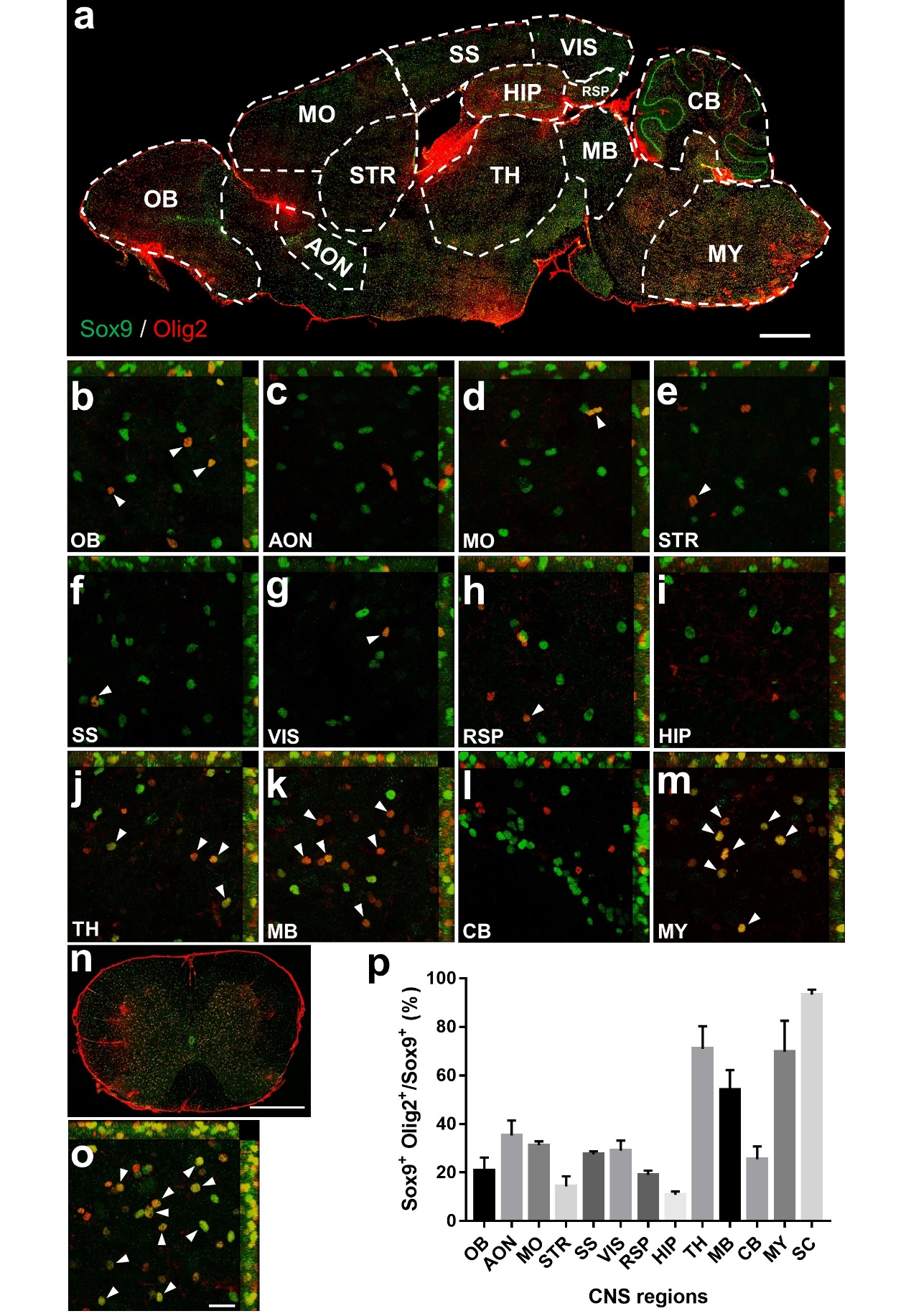
**

(a) Sagittal overview of the mouse brain indicating a variety of brain regions of interest. (b-m) 60x images showing the olig2 and Sox9 double immuno-staining in a variety of mouse brain regions of interest. The white arrowheads indicate Sox9-labeled astrocytes that express Olig2. In the OB (b), AON (c), STR (e), HIP (i), CB (l) and the cortical areas including the MO (d), SS (f), VIS (g) and the RSP (h), a relative low proportion of astrocytes (＜40%) express Olig2. In the MB (k), around half of the astrocytes express Olig2, while in the TH (j) and the MY (m), many more astrocytes (~70%) have Olig2 immune reactivity. (n) Coronal overview of the mouse SC indicating Olig2^+^ astrocytes. (o) The co-localization of Olig2 and Sox9 in the SC indicate that a majority of the astrocytes（93.3%）express Olig2. (p) The proportion of Olig2^+^ cells among all of the Sox9^+^ cells in different CNS regions. Data are represented as mean ± SEM. Scale bars: 1000 μm (a), 20 μm (b-m, o), 500 μm (n).
